# Efficacy of team-based collaborative care for distressed patients in secondary prevention of chronic coronary heart disease (TEACH): study protocol of a multicenter randomized controlled trial

**DOI:** 10.1186/s12872-020-01810-9

**Published:** 2020-12-10

**Authors:** Christoph Herrmann-Lingen, Christian Albus, Martina de Zwaan, Franziska Geiser, Katrin Heinemann, Martin Hellmich, Matthias Michal, Monika Sadlonova, Ralf Tostmann, Rolf Wachter, Birgit Herbeck Belnap

**Affiliations:** 1 Department of Psychosomatic Medicine and Psychotherapy, University of Göttingen Medical Center, Von-Siebold-Str. 5, 37075 Göttingen, Germany; 2 German Center for Cardiovascular Research (DZHK), Partner Site Göttingen, Göttingen, Germany; 3 Department of Psychosomatics and Psychotherapy, University of Cologne, Cologne, Germany; 4 Department of Psychosomatic Medicine and Psychotherapy, Hannover Medical School, Hannover, Germany; 5 Department of Psychosomatic Medicine and Psychotherapy, University of Bonn Medical Center, Bonn, Germany; 6 Institute of Medical Statistics and Computational Biology, Faculty of Medicine, University Hospital Cologne, University of Cologne, Cologne, Germany; 7 Department of Psychosomatic Medicine and Psychotherapy, University Medical Center Mainz, Mainz, Germany; 8 German Center for Cardiovascular Research (DZHK), Partner Site Rhine-Main, Mainz, Germany; 9 Department of Thoracic and Cardiovascular Surgery, University of Göttingen Medical Center, Göttingen, Germany; 10 Clinical Trials Unit, University of Göttingen Medical Center, Göttingen, Germany; 11 Clinic and Policlinic for Cardiology, University Hospital of Leipzig, Leipzig, Germany; 12 Center for Behavioral Health, Media, and Technology, University of Pittsburgh School of Medicine, University of Pittsburgh, Pittsburgh, PA USA

**Keywords:** Coronary heart disease, Secondary prevention, Psychological distress, Blended collaborative care, Randomized controlled trial

## Abstract

**Background:**

Coronary heart disease (CHD) is the leading cause of death and years of life lost worldwide. While effective treatments are available for both acute and chronic disease stages there are unmet needs for effective interventions to support patients in health behaviors required for secondary prevention. Psychosocial distress is a common comorbidity in patients with CHD and associated with substantially reduced health-related quality of life (HRQoL), poor health behavior, and low treatment adherence.

**Methods:**

In a confirmatory, randomized, controlled, two-arm parallel group, multicenter behavioral intervention trial we will randomize 440 distressed CHD patients with at least one insufficiently controlled cardiac risk factor to either their physicians' usual care (UC) or UC plus 12-months of blended collaborative care (TeamCare = TC). Trained nurse care managers (NCM) will proactively support patients to identify individual sources of distress and risk behaviors, establish a stepwise treatment plan to improve self-help and healthy behavior, and actively monitor adherence and progress. Additional e-health resources are available to patients and their families. Intervention fidelity is ensured by a treatment manual, an electronic patient registry, and a specialist team regularly supervising NCM via videoconferences and recommending protocol and guideline-compliant treatment adjustments as indicated. Recommendations will be shared with patients and their physicians who remain in charge of patients’ care. Since HRQoL is a recommended outcome by both, several guidelines and patient preference we chose a ≥ 50% improvement over baseline on the HeartQoL questionnaire at 12 months as primary outcome. Our primary hypothesis is that significantly more patients receiving TC will meet the primary outcome criterion compared to the UC group. Secondary hypotheses will evaluate improvements in risk factors, psychosocial variables, health care utilization, and durability of intervention effects over 18–30 months of follow-up.

**Discussion:**

TEACH is the first study of a blended collaborative care intervention simultaneously addressing distress and medical CHD risk factors conducted in cardiac patients in a European health care setting. If proven effective, its results can improve long-term chronic care of this vulnerable patient group and may be adapted for patients with other chronic conditions.

*Trial registration*: German Clinical Trials Register, DRKS00020824, registered on 4 June, 2020; https://www.drks.de/drks_web/navigate.do?navigationId=trial.HTML&TRIAL_ID=DRKS00020824

## Background

A recent survey estimates coronary heart disease (CHD) prevalence among Germans aged 40–79 years at 9.3% [1]. Despite advances in treatment, CHD is still the leading cause of death and years of life lost in Germany and worldwide, causing the largest burden of disability (DALY) [2]. In Germany it produces per year 650,000 hospitalizations and overall costs of € 6.8 billion [3]. Psychosocial distress is a major risk factor in > 25% of patients with CHD [4] impairing health-related quality of life (HRQoL), healthy lifestyles, and treatment adherence [5, 6], and thereby increasing mortality and morbidity [7].

While excellent treatments and rehabilitation programs are available for acute CHD, patients’ long-term prognosis and HRQoL are closely tied to their subsequent health behaviors [7]. However, patients and their families are often left unsupported in the task of daily managing the chronic stage of CHD (e.g., coping with illness, adhering to lifestyle changes and medication regimens), and current programs for behavior change (rehabilitation, disease management) have not shown the desired long-term effects, mainly due to lack of: timely follow-up, attention to patient preferences, and coordination with their treating physicians [8].

A meta-analysis has shown that in the U.S. depression collaborative care (CC), a team-based care strategy exclusively focusing on mood, is effective in improving depressive symptoms in CHD patients [9]. Consequently, current European guidelines recommend CC for CHD patients with coexisting depression or anxiety [7]. However, focusing primarily on depression treatment seems to confer no clear somatic benefit [9, 10]. We searched pertinent databases for published and ongoing trials providing collaborative care to CHD patients. Most relevant to our research question, the U.S. TeamCare trial, targeting both mental health and cardiovascular risk factors, showed that this “blended” CC strategy (TeamCare [TC]) can improve both conditions [11], at no or minimal extra cost [12].

In a pilot study in Göttingen (n = 40) we tested the feasibility and acceptance of a TC approach in patients with CHD in the German health care system [13, 14] For the first time in Germany, we combined the theoretical framework from U.S. trials [11, 15–17] with our clinical expertise from multiprofessional psychosomatic, family medicine, and cardiac rehabilitation facilities. Our pilot data showed that a brief six-month intervention significantly reduced distress and cardiac risk factor burden and 83% of patients rated their satisfaction with the study intervention as high or very high [13, 14].

## Methods/design

### Study design and objectives

TEACH is a confirmatory, randomized, controlled, two-arm parallel group, observer blind, interventional (behavioral) trial. If positive, it will confirm the efficacy of a multidisciplinary, team-based intervention addressing both medical and psychosocial risk factors delivered via the collaborative TeamCare strategy. This will close the gap in comprehensive long-term follow-up care for patients burdened by both chronic CHD and psychosocial distress by improving their HRQoL and overall treatment adherence, which then can reduce risk factor burden, morbidity and mortality.

**Fig. 1:**
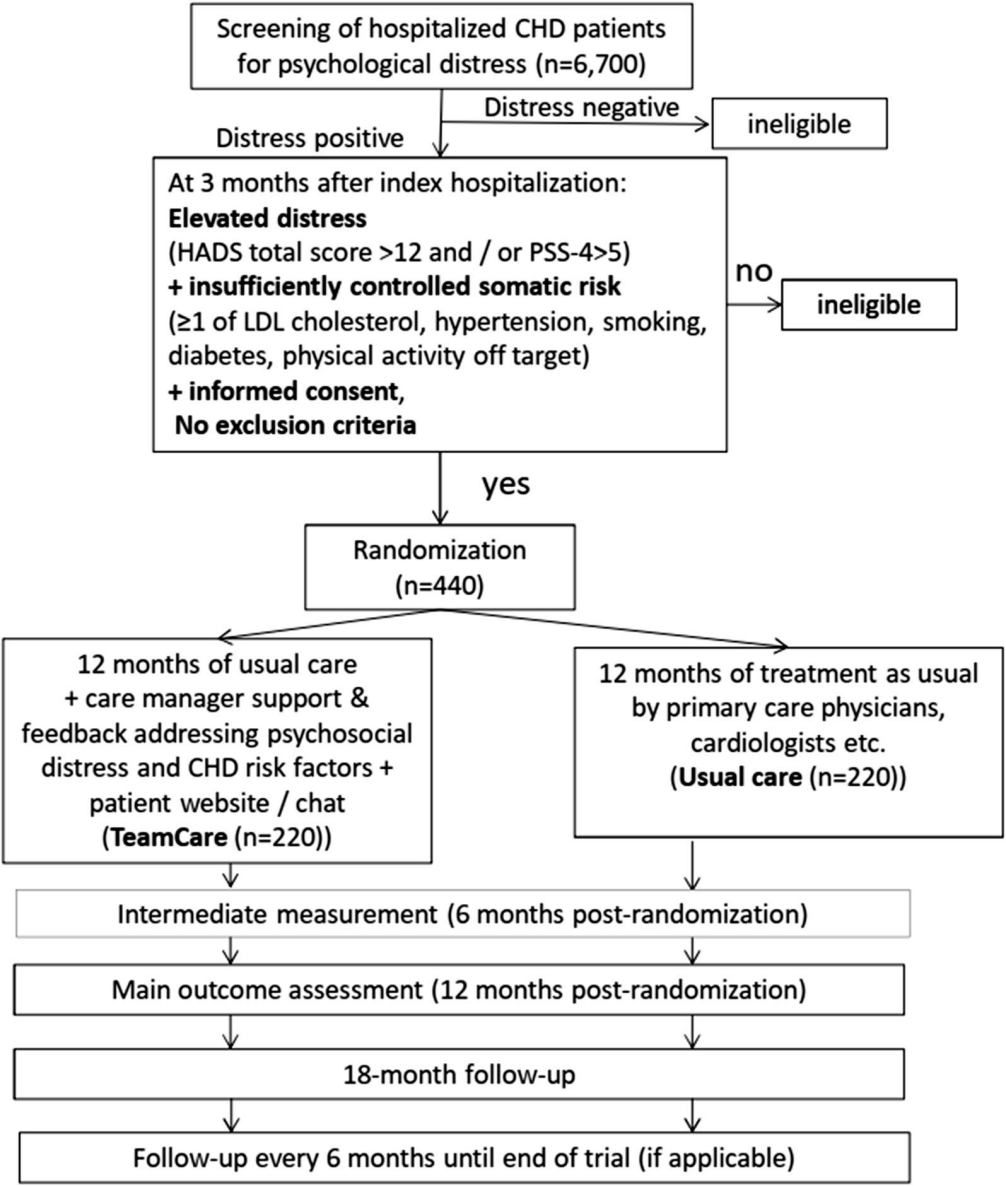
Trial flow. *CHD *coronary heart disease, *LDL * low density lipoprotein, *HADS *hospital anxiety and depression scale; *PSS-4 *perceived stress scale (4-item version)

We will randomize 440 psychosocially distressed CHD patients with at least one insufficiently controlled somatic CHD risk factor to 12 months of either (a) their physicians’ usual care (UC); or (b) UC plus TC for both distress and CHD risk factors (Fig. 1).

Our primary hypothesis is that TC for both CHD and psychosocial distress will improve patients’ HRQoL more than their physicians’ UC for these conditions. Secondary hypotheses are that TC will improve health behaviors, treatment adherence, cardiac risk factors, mood and anxiety symptoms, and health care utilization.

### Study setting and sample

We recruit from the following six German University Hospitals: Göttingen, Cologne, Bonn, Hannover, Leipzig, and Mainz, where we screen hospitalized patients aged 18–85 with CHD for psychological distress, and those who screen positive will remain eligible. Since many patients will receive cardiac rehabilitation after hospital discharge for typically 3–4 weeks, we expect that their cardiac risk factors will initially be controlled and that their distress levels will improve. However, after returning to their home environment, they are challenged with implementing health behaviors and treatment adherence into their daily routines. We will therefore re-screen all screen-positive patients three months later to ensure that they still report psychological distress, have at least one uncontrolled cardiac risk factor, and meet all other eligibility criteria. Those who continue to be eligible and give their written consent are randomized 1:1 into one of the study groups (N = 440).

#### Eligibility screening

We will use a stepwise screening and enrollment procedure.

##### Step 1: Screening at the hospital (Screen 1)

Our study nurses approach cardiac units personnel of our study hospitals to inquire about any new admissions for CHD who may be appropriate for our study. In keeping with the privacy rules, they ask hospital staff who have routine clinical contact with identified patients to request patient’s’ verbal agreement to be approached before entering their room. Study nurses then provide a brief description of our study and seek their written informed consent to undergo our screening procedure. Patients meeting all inclusion and with no exclusion criteria are then administered the 14-item Hospital Anxiety and Depression Scale (HADS) [18] and the 4-item Perceived Stress Scale (PSS-4) [19] to establish their level of psychological distress (Table 1).

**Table 1:** Inclusion/exclusion criteria

*Inclusion criteria for screening procedure*
1. Patients of any gender
2. Ages 18–85 years
3. Hospitalized in a cardiology or cardiothoracic surgery department with a CHD diagnosis documented as:
(a) coronary angiography (> 50% stenosis in ≥ 1 major coronary vessel)
(b) and/or confirmed acute coronary syndrome
(c) and/or history of coronary revascularization (percutaneous intervention or coronary artery bypass surgery)
4. Ability to speak, read and understand German
5. HADS > 12 and/or PSS-4 > 5
6. Signed informed consent to screening procedure
*Inclusion criteria for main trial*
1. HADS > 12 and/or score on PSS-4 > 5
2. > = 1 insufficiently controlled cardiac risk factor as defined as:
(a) Hypertension with blood pressure > = 140/90 mmHg
(b) Hyperlipidemia with LDL-cholesterol > = 70 mg/dl
(c) Current smoking
(d) Diabetes with HbA1c > = 7.0%
(e) Physical inactivity: self-report of < 150 min. of moderate or < 75 min. of vigorous physical activity per week
3. Written informed consent for study participation
*Exclusion criteria*
1. Severe cognitive impairment, defined as known dementia or inability to follow the assessment instructions
2. Communication difficulties (e.g. hard of hearing, aphasia)
3. Acute or severely disabling non-cardiac disease with estimated survival < 1 year
4. Need for more specialized cardiac or mental health interventions or structured rehabilitation programs such as
(a) recurrent ACS or coronary surgery after the index hospitalization
(b) severe mental disorders (e.g. acute psychoses) or addiction (except tobacco)
5. Participation in another treatment trial likely to affect the outcomes of interest or interfering with the trial procedures

*ACS* acute coronary syndrome, *CHD* coronary heart disease, *HADS* Hospital Anxiety and Depression Scale, *HbA1c* Haemoglobin A1c, *LDL* low density lipoprotein, *PSS-4* short version of Perceived Stress Scale

All eligible patients receive a brief study brochure and we inform hospital physicians of patients’ HADS/PSS-4 scores, so that they can start treatment, if indicated.

##### Step 2: Screening for the study (Screen 2)

After hospital discharge many German patients with CHD are enrolled in a rehabilitation program that is in most cases delivered on an inpatient basis and typically lasts several weeks. In these programs patients are instructed in general health behaviors, but often struggle to implement these in their daily routines afterwards. Hence, many continue to be at-risk for a decline in their health [8]. Therefore, we contact all patients who screened positive in hospital (Screen 1) and rescreen them via telephone to confirm that they still have at least one uncontrolled cardiac risk factor and continue to be at least moderately distressed and meet our other study eligibility criteria. If the risk factor level cannot be determined by patient self-report, we will obtain the relevant information from medical records and/or treating physicians. All patient who continue to be eligible (see Table 1) and are interested in participation in our study are then asked to come to the recruiting center.

### Baseline assessment and randomization

All patients eligible for the study are invited to come to the respective recruitment center, where they sign their study consent and complete their baseline assessment. A study physician assesses patients’ detailed medical history, performs a basic medical exam (for details see Additional file 1), and records their prescribed medications (Table 2). Afterwards a study member administers the psychometric assessment. All data are pseudonymized and directly entered into our certified central SecuTrial data base via password-protected tablet computers, that are programmed with skip patterns, drop-down menus, check-off boxes, and error checking routines monitoring forms for out-of-range values and missing data. A data safety plan has been developed for ensuring data protection.

**Table 2:** Visit schedule and assessments

Periods	Name	Screen 1 (in-hospital)	*Screen 2* (telephone)	*Randomization*	Treatment		*Follow-up*		
Visits				Baseline Assessment (Visit)	6 months (telephone)	EOT (visit)	FU 18 (visit)	FU 24^a^ (telephone)	FU 30^a^ (telephone)
	Time	Day-120–day-90	Day-14	Day 0	Month 6	Month 12	Month 18	Month 24	Month 30
informed screening consent		X							
Screen 1: Inclusion and exclusion criteria		X							
Screen 2: Inclusion and exclusion criteria			X						
Informed study consent				X					
Demographics				X		X	X		
Medical History^b^				X	X	X	X		
Physical examination^c^				X		X	X		
Laboratory tests^d^				X		X	X		
Psychometric battery 1^e^				X	X	X	X	X	X
Psychometric battery 2^f^				X		X	X		
Medication				X	X	X	X	X	X
Actimetry				X		X	X		
Adverse Events/Vital Status				X	X	X	X	X	X

*EOT* end of study treatment, *FU* follow-up

^a^FU 18 will be administered in all participants to measure treatment durability. FU 24 and FU 30 will only be administered before the end of study, i.e. only in participants included during the first 12 or 6 months, of recruitment, respectively

^b^Full medical history and demographics will be obtained at baseline only. Only relevant changes from baseline will be documented during later assessments

^c^Physical examination will typically be limited to heart rate, blood pressure, body height (baseline only) and weight. Other physical examinations will only be performed based on clinical need

^d^Laboratory tests comprise LDL cholesterol, HbA1c, creatinine, and cotinine

^e^Questionnaires included: HeartQoL (primary outcome), HADS, PSS-4

^f^Questionnaires included: see Table 4

#### Randomization

If patients continue to meet our study eligibility criteria following the baseline assessment and are still interested in participating in the study, our data management system automatically assigns patients in a 1:1 ratio stratified by study site based on permuted blocks of varying size to one of two treatments: (1) physicians’ usual care plus TC for both CHD and psychosocial distress; or (2) physicians’ usual care only (Fig. 1). Then the study personnel informs the patients of their group assignment, and if randomized to TC, they schedule the first intervention telephone call. Afterwards the project coordinator sends a letter to patients’ treating physicians to inform them of their study participation and group allotment.

### Study treatments

#### Team care intervention

Our TC intervention is a stepwise patient-centered treatment strategy that promotes proactive, timely follow-up and support by a specifically trained nurse care manager (NCM). Intervention manual and NCM training materials are adapted from materials published in earlier studies [e.g. 11] or developed during our own previous studies [14, 15, 17]. Each recruitment center assigns at least one NCM to the intervention cohort recruited at that site. NCMs contact their patients during regular telephone or video calls to promote health behaviors crucial for CHD secondary prevention and impart skills to cope with distress.

During their first contact, the NCM assesses patient’s treatment preferences and discusses in a shared-decision making manner an individual care plan and its implementation into daily routine (e.g., blood pressure control, stress reduction, exercise). Over the course of 12 months NCMs will then support patients by: (a) monitoring symptoms, risk factors and treatment adherence; (b) assessing vitals (by self-report or direct measurement), medications, and behavioral goals and progress; (c) identifying obstacles in implementing behavior change and offering solutions; (d) connecting patients with external resources (specialists, programs for supervised exercise, smoking cessation etc., or self-help groups) to improve self-management; and (e) offering care coordination across health care providers in close collaboration with patients’ treating physicians. NCMs use psychoeducation and motivational interviewing techniques to promote patients’ adherence with care plans (Table 3).

**Table 3:** Overview of treatment components

Treatment components	Team-care	Usual care
*Patient*		
Informed of risk factor status	** + **	** + **
Informed of randomization status	** + **	** + **
Regularly contacted by nurse care manager over 12 months	** + **	
Receives personalized text messages to support healthy behavior	** + **	
Gets access to website offering educational materials and supervised chatroom	** + **	
*Nurse care manager*		
Phones patients at regular intervals over 12 months to provide support for managing distress and CHD risk		
(a) basic education re: CHD and treatment goals;	** + **	
(b) psychoeducation re: distress and mental health;	** + **	
(c) links to web resources and review lessons;	** + **	
(d) motivational interviewing and problem-solving techniques as needed	** + **	
(e) encourages and reviews health behaviors (smoking cessation, diet exercise, sleep, relaxation, medication adherence, etc.).;	** + **	
(f) confirms use of guideline-recommended treatments to target	** + **	
(g) promotes adherence/adjustment of pharmacotherapy in concert with patient’s treating physician;	** + **	
(h) promotes self-monitoring of BP, diet, doctor visits & reviews results;	** + **	
(i) monitors for treatment response, relays information to treating physician;	** + **	
(j) screens for suicidal ideation and cardiac instability	** + **	
(k) suggests referrals to cardiology, psychotherapy, self-help as appropriate	** + **	
Sends individualized text message reminders between calls	** + **	
Moderates web chatroom	** + **	
Meets with TCT in weekly videoconferences to discuss patient progress and treatment recommendations	+	
Relates TCT recommendations to treating physicians for consideration	+	
*Treating physicians*		
Informed of their patients’ baseline risk factor status and treatment assignment	+	+
Provide care for their patients’ distress and CHD risk	+	+
Can initiate, adjust, or discontinue pharmacotherapy	+	+
Receive regular progress reports from the care manager	+	
Receive TCT recommendation for treatment adjustment	+	
Offered assistance with patient referral to specialist care or self-help groups etc	+	
Informed if medical or mental condition significantly worsens (e.g., chest pain, suicidality)	+	( +)*

*BP* blood pressure, *CHD* coronary heart disease, *TCT* TeamCare Team

* Treating physicians of UC patients will be informed of worsening condition observed during blinded assessments only

Starting with existing educational materials developed for our pilot study, we also provide a patient website with relevant audiovisual health information and regularly featured tips in support of behavioral change and stress reduction that is open to all participants and their family members. Furthermore, in a password-protected internet chat room for intervention participants only we offer weekly moderated text chats with an NCM, psychologist, or TC Team (TCT) member to obtain emotional support and learn from other patients’ experiences. At the end of each weekly session, we close the chat function, but all chats remain accessible to intervention participants in read-only mode. Participants are advised to choose nicknames and not disclose any identifiable information, which we will monitor during the chats. To further enhance the TC support, we send individualized text messages and recommendations for freely available mobile applications to remind patients of desired activities (such as exercising, taking medications, etc.) and encourage adherence to the treatment plan.

During each patient contact NCMs enter all information into our electronic patient registry (EPR) that will also guide the NCM through the contacts and prompt for relevant questions based on our manual. Based on own experiences from the U.S.A. (BHB) we programed the EPR so that NCMs can easily sort their patient panel by: last contact, symptom level, risk factors, and goal progression, and will be alerted to treatment gaps or deterioration. During the first 3–4 months of the intervention phase, NCMs contact patients twice a month for typically 30 min on average, after which contacts are once a month, unless different intervals are indicated.

##### Case review

We hold weekly case review meetings, in two groups of 3 local NCMs each, via videoconference, where the patient panel and records are projected from the EPR. At case review meetings, each NCM presents their patients to a central TCT, consisting of a cardiologist/internist and mental health specialist. With the aid of the EPR sort function the TCT can readily review all newly randomized participants and those who continue to show insufficiently controlled risk factors or no progression in their goals. Periodically, a random sample of all patients will be discussed as a quality control measure.

The TCT (a) monitors that patients receive evidence-based treatment for both conditions, (e.g., “classical” CHD risk factors, depression); (b) makes guideline-based treatment recommendations, if necessary; and (c) assists the NCMs in addressing treatment barriers. Recommendations may include but are not limited to (i) brief behavioral interventions delivered either by the NCM, through electronic media (e.g., our study website), or bibliotherapy, (ii) initiation or adjustment of medications by the treating physician, (iii) referral to external resources (Table 3).

Following each case review, the NCMs contact their patients to relay the treatment recommendations and later to assess the clinical response. The NCMs also work closely with the patients’ treating physicians and ensure that patients keep all appointments, pick up their prescriptions, and follow other treatment agreements. The NCMs regularly update the treating physician(s) about the patient’s treatment progress, TCT recommendations, and alert him/her, if any concerning or safety issues arise. The treating physician remains the primary care provider who adjusts the treatment including adjustment of medications and/or referral to specialty cardiac or mental health care, while the NCM supports the treatment implementation (Table 3).

#### Usual care (UC)

A study member informs all participants randomized to the UC group of their assignment status at the conclusion of their in-person baseline assessment and provides a leaflet summarizing their individual psychosocial, behavioral, and medical risk factor status together with relevant sections of the current German patient guideline for chronic CHD patients [20]. They also send a letter to their treating physician indicating their patients’ study assignment and encouraging a follow-up appointment to discuss the patient's insufficiently controlled cardiac risk factors and elevated level of distress. Beyond that, UC patients continue to receive care at the discretion of their physicians (Table 3).

### Follow-up assessments and outcomes

At 12- (end of treatment) and 18-months after randomization participants are invited to come to their recruitment center, where a study physician and a study assessor blinded to participants’ randomization status administer the medical and psychometric assessment batteries. All patients are followed for a minimum of 18 months, 6 months after the completion of our TC, in order to document sustainability of our intervention. In addition, centrally located, blinded assessors will administer self-report psychometric and medical questionnaires via telephone or video call 6 months after randomization. and every 6 months after the 18-month assessment until the end of the trial, if applicable (Table 2). Study personnel will enter all data collected during assessments directly into our SecuTrial database as described in 2.3.

At every in-person assessment (baseline, 12-, 18-months) we will collect blood and urine samples to determine risk factors, such as LDL HbA1c, and cotinine. Blood and urine samples will be centrifuged, aliquoted and stored at − 80 °C for central analysis before the end of the trial. Samples may be stored for up to ten years and will be disposed of afterwards. Details on the biological specimen handling, storage and shipping are described in our Biological Specimen Handling Manual.

To guide the TC treatment, open assessments of risk factors are performed for intervention patients at baseline and at least once during the intervention period, either by review of routine medical assessments by treating physician(s) or—if no current records are available—by the study center.

#### Primary outcome

Quality of life is a widely recognized outcome, especially for chronic conditions. Indeed, recent German treatment guidelines for CHD patients recommend HRQoL as a primary outcomes goal [21], and we confirmed in a meeting with a local self-help group of cardiac patients that HRQoL coincides with their preference as an outcome. Furthermore, as there is not a single measure that assesses both our target conditions, CHD and distress, HRQoL can serve as a global measure of health. Therefore, our primary hypothesis is that compared to UC more patients in our TC intervention group will report a clinically meaningful improvement in HRQoL at 12-months.

The HeartQoL questionnaire [22] assesses HRQoL for patients with CHD, thus allowing to measure overall treatment response for our multimorbid population. It is a 14-item self-rating instrument that is proven reliable, valid, and responsive to change across various CHD populations, including the German version used in our study [23]. In the survey from de Smedt et al. [23], 7,449 patients completed the HeartQoL questionnaire with an excellent internal consistency for the global HeartQoL scale (Cronbach's alpha α = 0.92) and the physical subscale (α = 0.91), as well as good internal consistency for the emotional subscale (α = 0.87). We defined response to treatment as ≥ 50% increase in HeartQoL score (mean of 14 items scored 0–3, range 0–3) (i.e. (score_12 months_-score_baseline_)/(3-score_baseline_) ≥ 50%).

#### Secondary outcomes

Our main secondary outcome measure is a composite medical risk factor score based on current European Society of Cardiology (ESC) cardiovascular prevention guidelines [7]. Composite risk factor scores have been used in previous intervention research aiming to reduce risk factor burden (e.g., [11, 59]), and different ways for computing such scores (e.g. by weighting) have been proposed. Established scores such as the ESC SCORE [7] or the PROCAM score [24] have only been validated for individuals without known CHD, thus rendering all CHD patients as uniformly high risk. Therefore, Katon et al. [11] computed a composite measure by means of a multivariate scaled marginal model, where each outcome measure is scaled by its standard error. However, that model requires continuous data and does not seem to be feasible for the risk factors of interest in our trial. We will therefore use dichotomous data based on either presence vs. absence of a risk factor (e.g. smoking) or sufficient vs. insufficient control according to the ESC guideline. In accordance with Khaw et al. [25], our score will be computed as a sum (range 0–5) of the following most important, modifiable but often insufficiently controlled CHD risk factors: (1) Blood pressure > = 140/90 mmHg; (2) LDL-cholesterol > = 70 mg/dl; (3) current smoking; (4) HbA1c > = 7.0%; (5) physical inactivity as defined: < 150 min. of moderate or < 75 min. of vigorous physical activity per week.

Additional secondary endpoints include change in body mass index, medical events, health care utilization, and use of evidence-based medications. Disease-relevant health behaviors (e.g., physical activity, smoking, medication adherence) are assessed by self–report and objective measures (e.g. electronic activity trackers, cotinine in urine samples).

To further examine the impact of distress and mental health on patients’ outcomes, we will (a) explore patients’ change in HADS and PSS-4 scores across the time from hospitalization to end of study and (b) further assess their psychosocial risk and well-being with validated instruments (Table 4).

- HADS
- The Hospital Anxiety and Depression Scale (HADS) [18, 26] assesses the severity of anxiety and depressive symptoms in physically ill patients. It is a self-rating instrument on a four-step Likert-Scale with 14 items, with higher scores signifying increased severity. The German version has been validated in several large samples of cardiac patients, mainly with coronary artery disease (e.g. [27–29]), is equivalent to the original English version [26], and norms are available for these patients. In a review of 747 studies using the HADS for different purposes, Bjelland et al. [30] found a mean Cronbach’s alpha of 0.82 for the subscale “anxiety” and of 0.83 for the subscale “depression”. For the inclusion criteria of elevated distress in our study, we define the relevant cut-off at a total score > 12 (range 0–21), which is expected to detect even mild symptomatology as this has already been shown to be prognostically relevant [18].
- PSS-4.
- The PSS-4 [31] is the short version of the well-established Perceived Stress Scale (PSS; [32]) that measures subjectively perceived distress. To assure equal validity in the German sample, the items are drawn from the German version of the PSS-10 [33], which yielded good psychometric properties equivalent to the original version. The items are answered on a 5-step frequency scale (range 0–16), where a higher score reflects the respondent’s perception that the demands exceed his/her ability to cope. For the present study we define a relevant screening cut-off score > 5 for defining elevated distress as we did in our pilot study [14].
- All outcomes will be assessed in all participants at baseline, month 12 (primary endpoint) and month 18. Selected self-report variables will additionally be assessed at month 6, and later until the end of the trial, i.e. after 24 months and 30 months in patients included during the early phase of the trial to test sustainability. We will track cardiac events and all-cause mortality by self-report, discharge notes, and death certificates, and document hospitalizations, numbers of outpatient visits, medication, and disability/return to work or early retirement for later cost-effectiveness analyses.

**Table 4:** List of assessment instruments

Outcome	Measurement	Description	References
Social support	ENRICHD Social Support Inventory (ESSI)	5-Item scale to assess perceived emotional social support in CHD patients	[34, 35]
Health Literacy	European health literacy survey (HLS-EU-Q16)	16 items to assess knowledge, motivation and competences to access, understand, appraise, and apply health information; 3 subscales: health promotion, health treatment and prevention	[36, 37]
Attachment	Experience in close relationship instrument revised (ECR-RD8)	8 items on a 7-step match scale with two subscales: attachment-related anxiety and avoidance	[38, 39]
Resilience	Resilience Scale (RS-13)	13-item short version to measure the ability of mental resistance	[40, 41]
Demoralization	Demoralization scale (D-S)	24-Item questionnaire with 5 subscales: loss of meaning and purpose, dysphoria, disheartenment, helplessness and sense of failure	[42, 43]
Heart-related fears	Herzangstfragebogen (HAF-17)	17-Item questionnaire assessing behavior and emotions regarding heart-related symptoms, German adaptation of Cardiac Anxiety Questionnaire [44]	[44–46]
Angina symptoms	Seattle Angina Questionnaire (SAQ)	Assessing in 5 subscales: disease perception, physical limitation, angina frequency, angina stability and treatment satisfaction	[47, 48]
Medication adherence	Rief Adherence Index (RAI)	Describes frequency of their general past and present behaviors concerning medication intake as a percentage range on a five-stage Likert scale	[49]
Therapeutic alliance	Working Alliance Inventory—short revised (WAI-SR-P)	Measures the therapeutic alliance between patient and therapist from patient view. We use to measure the alliance with NCM in TC group. 12-items assess 3 subscales: binding, process, goals	[50, 51]
Physical activity	International Physical Activity Questionnaire (IPAQ-7)	7-Item to self-record hours and days of various activities with strenuous or moderate physical activity over7-day period	[52]
Personality functioning	Operationalized Psychodynamic Diagnosis—Structure Questionnaire short form (OPD-SFK)	12-Items assessing elements of personality structure	[53, 54]
Treatment satisfaction	Purpose-designed questions	Questions about overall treatment satisfaction, satisfaction with treatment of heart disease, satisfaction with treatment of distress	

### Trial status

Prescreening for the TEACH Trial has started in August 2020 and enrollment in the main trial has started in November, 2020.

## Data safety monitoring

An independent Data Safety Monitoring Committee (DSMC) reviews a defined data set in regular intervals and evaluates whether patient safety is ensured and whether the conduct of the trial complies with ethical requirements. Regular on-site monitoring by the UMG Clinical Trials Unit ensures compliance of study conduct with all applicable regulatory and ethical standards. The work of the DSMC is governed by a DSMC charter.

Furthermore, the study coordinating team will generate up-to-date administrative reports by the data management system to monitor: (1) trial enrollment by study center; (2) NCMs’ caseloads; (3) rates of follow-up assessments; (4) missed study assessments so that patients may be recontacted; (5) patients’ clinical status for data safety monitoring purposes; and (6) potential protocol deviations that the study investigators review at staff meetings as appropriate.

## Adverse events

Our blinded assessors inquire routinely about any adverse events (e.g. emergency room visits, hospitalizations, mental health visits) participants may have experienced since their past assessment. Since TEACH does not test any medicinal product or medical device, we decided to define attempted suicide, acute coronary syndrome, or death as serious adverse events of special interest requiring immediate notification of the coordinating investigator. Adverse events reported during care management are also documented.

In case the participant’s adverse event warrants medical attention, the assessors and NCMs will follow our standard operating procedures to initiate emergency care, to encourage participants to seek professional help, or to provide information about local emergency care or mental health resources.

All severe adverse events of special interest are reviewed by the coordinating investigator and reported to the DSMC.

An insurance will cover any accidents occurring to patients while attending scheduled trial visits.

## Statistical analysis

The primary analysis is based on the intention to treat with no exclusions (full analysis set). The odds of responding to treatment (i.e. ≥ 50% increase in HRQoL from baseline to month 12) are compared between treatment groups (TC vs. UC) by the Mantel–Haenszel test stratified by study site. Heterogeneity of the common odds ratio over strata is evaluated by the Breslow-Day test. A missing response status is counted as failure. Numbers needed to treat are derived from the Mantel–Haenszel odds ratio. Analysis of all subjects who were essentially treated and observed per protocol (PP set) is supportive.

Moreover, the main outcome measure “change in HeartQoL global scale from baseline to month 12” is evaluated by mixed model for repeated measures (MMRM) with fixed effects baseline, study site, treatment, time and the interaction treatment*time (ARH1-structured covariance matrix over time) and estimation of corresponding marginal means and treatment contrasts. It will be expressed in absolute values and in relation to minimal clinically important differences (MCID). The possibly moderating influence of study site, age, sex, disease severity (number of diseased coronary arteries, history of myocardial infarction, CCS and NYHA classes, baseline distress level), and risk factor burden is explored. Since mixed models can be expected to yield valid results only in case of missingness-at-random, multiple imputation approaches are taken to assess the robustness of the results. Specifically, missing values due to death, illness, or chance are separately imputed assuming mixtures of missingness-not-at-random patterns. Imputation data sets are post-processed by multiplication with factors and addition of offsets (tipping point analysis [55]). The influence of clustering of subjects by care providers (particularly in TC) is investigated by multilevel modeling [56].

Secondary outcomes are analyzed along the same lines. The count of present dichotomous risk factors over time is analyzed by means of generalized estimating equations (GEE). Moreover, a resampling approach for the analysis of correlated multiple endpoints is taken, e.g. as recently described by Ristl et al. [57]. Time-to-event (e.g., survival) distributions are summarized by the Kaplan–Meier method and compared by the (stratified) log-rank test. Since mortality rates will be low only small survival differences are expected to emerge during the trial. A subgroup analysis will be done by sex (expected male to female ratio about 2:1 [11]). Adverse events will be aggregated by category (e.g., MedDRA) and listed.

### Sample size calculation

Katon et al. [11] observed in their TeamCare Trial proportions for the response to treatment of 60% (intervention group) and 30% (control group), respectively, for ≥ 50% decrease in depression score. Thus, assuming a ≥ 50% increase in HRQoL on the HeartQoL score, we expect response proportions of 50% (TC) and 35% (UC), i.e. a difference of 15% absolute. The chi-square test requires 170 subjects per group to detect this difference with a power of 80% and a two-sided type-I-error of 5% (Stata/SE 16.1, StataCorp LLC, College Station, TX, USA). Similar to Katon et al. [11] we expect a standardized effect of at least 0.3 in favor of TC. The two-sample *t*-test requires 176 subjects per group to reach an 80% power at a two-sided type-I-error of 5%. Accounting for 15% attrition and (partial) therapist clustering [56] in TC (+ 5%), 220 (≈176/0.8) patients need to get randomized per group, i.e. 440 subjects in total. Adjusting for baseline in a mixed model for repeated measures (MMRM) approach is likely to further increase the statistical power.

## Discussion

One of the major challenges in chronic care is the sustainable implementation of complex treatment regimens for patients with multiple chronic diseases. Indeed, 80% of patients with CHD, one of the most prevalent chronic conditions, report three or more chronic conditions, substantially impacting their HRQoL [58]. Specifically patients with comorbid psychosocial distress are especially challenged with the implementation of healthy behaviors into their daily lives [59]. Although collaborative care programs have been shown effective in improving HRQoL and symptoms in chronic conditions, it would be impractical to deploy separate collaborative care treatments for each condition.

TEACH is the first randomized clinical trial to evaluate the effectiveness of a “blended” strategy of collaborative care (TC) for treating both CHD and emotional distress in a European health care system. If proven effective, it will not only improve care for distressed patients with CHD, but serve as a benchmark for a treatment strategy of other clusters of chronic medical conditions.

We designed our TC intervention to be easily integrated into routine care for CHD by: (1) screening patients in hospital by two brief and well accepted instruments; (2) confirm the positive screen 3-months after their hospital discharge as persistent distress increases risk in CHD patients; [60] (3) delivery of intervention via telephone or video calls; (4) providing regular follow-up and motivational support calls; (5) deploying a shared decision technique to allow for patients’ treatment preferences; (6) promoting delivery of guideline-recommended care for the target conditions; and (7) coordinating care across patients’ physicians. Our NCMs are supervised by a multidisciplinary team of study physicians and psychologists who discuss patients’ progress at weekly case review meetings and make treatment recommendations, if necessary that are related to patients’ treating physicians who remain responsible for their patient’s treatment. Innovative to our study design is the incorporation of a patients website to promote patients self-management by using its educational tools and exchange experiences with other patients in the weekly chat room. Furthermore, we will also use short text messages to remind patients of upcoming appointments or regular health behaviors (e.g., blood pressure check, weighing) to promote their adherence.

Our TEACH study design has several limitations. We focused our primary hypothesis on HRQoL and not on differences in morbidity or mortality. Nevertheless, we collect data on major clinical events to inform future larger trials to establish TC’s impact on “hard” clinical outcomes. Second, we require patients to have moderate levels of emotional distress (HADS > 12 or PSS > 5) as they have been shown to impact CHD (e.g., [61, 62]). However, patients with an even lower distress level may also benefit from an intervention. Third, as many CHD patients in the German health care typically participate in a rehabilitation program post hospital discharge, we rescreen patients after 3 months to identify those at continued risk, which may delay care for patients who would have benefitted from a more specific early intervention. Fourth, it will need to be tested how our intervention needs to be modified for use in other health care systems.

The TEACH trial will provide trial-derived evidence on whether a nurse-led and telephone-delivered “blended” collaborative care intervention (TC) for treating both CHD and emotional distress can be provided at scale at multiple German hospitals and is more effective at improving clinical outcomes than physicians’ standard care for these conditions.

## Supplementary Information

**Additional file 1**. Case Report Forms for the TEACH trial.

## Data Availability

Research data will be stored, managed and monitored at the UMG. The anonymized original data set will be published in a certified data repository for future use after completion of the trial and acceptance of the primary results publication. The study steering committee will form a subgroup for approving requests from external researchers to access the data.

## Abbreviations

AE: Adverse event
BMBF: Bundesministerium für Bildung und Forschung (German Ministry for Education and Research)
BP: Blood pressure
CC: Collaborative care
CHD: Coronary heart disease
DSMC: Data Safety Monitoring Commitee
EOT: End of treatment
EPR: Electronic patient registry
ESC: European Society of Cardiology
FU: Follow-up
HADS: Hospital Anxiety and Depression Scale
HbA1c: Haemoglobin A1c
HRQoL: Health-related quality of life
IMSB: Institute for Medical Statistics and Bioinformatics
LDL: Low density lipoprotein
NCM: Nurse care manager
PSS-4: Short version of perceived stress scale
RCT: Randomized controlled trial
TC: Team care (intervention)
TCT: TeamCare Team
UC: Usual care (standard medical care)
UMG: Universitätsmedizin Göttingen (University of Göttingen Medical Center)
WHO: World Health Organisation
