# Supplementary material for: Efficacy of team-based collaborative care for distressed patients in secondary prevention of chronic coronary heart disease (TEACH): study protocol of a multicenter randomized controlled trial
Source: BMC Cardiovasc Disord. 2020 Dec 10;20:520. doi: 10.1186/s12872-020-01810-9 (PMC7731481; doi:10.1186/s12872-020-01810-9)
Supplement: Supplementary file 1 — Additional file 1. Case Report Forms for the TEACH trial. [file 12872_2020_1810_MOESM1_ESM.pdf]

Pat. ID:	Site Code:	Date:

Only register Patient ID. if consent is given and case has been created in SecuTrial.

Patient identification and pre-screening in-hospital

1. Sex
   - ☐ male
   - ☐ female
   - ☐ diverse

2. Date of birth _ _ (mm) _ _ _ _ (yyyy) !stop in case of age <18y. or >85y.

3. Confirmed CHD, documented by at least one of the following criteria:

   ☐ Coronary angiography > 50% Stenosis in ≥ 1 main coronary vessel
   ☐ Confirmed acute coronary syndrome
   ☐ History of coronary revascularization, i.e. percutaneous intervention (PCI) or coronary bypass surgery; PCI or bypass surgery may have been performed during the current hospitalization.

4. Sufficient knowledge of German and reading skills (impression by visiting, if necessary in case of uncertainty ask the patient)
   ☐ no, then stop! ☐ yes

5. Severe comorbidities with an estimated survival of <1 year

   ☐ no ☐ yes, which?________________________________then stop!

6. Severe mental illness according to the patient record (e.g. acute psychosis, addictions other than tobacco)

   ☐ no ☐ yes, which?________________________________ then stop!

7. Severe cognitive impairment / dementia

   ☐ no ☐ yes, then stop!

8. Patient consent #1 (screening procedure) ☐ yes ☐ no, then stop!

9. HADS Score: _____________; PSS-4 Score: ______________

10. Patient possibly eligible ☐ yes ☐ no

11. Type of contact agreed upon ☐ by phone ☐ by E-mail

Pat. ID:	Site Code:	Date:

Screening (t-14) >=3 months after Prescreening

(Reassessment using the HADS and PSS4. Continue only if HADS>12 and/or PSS-4>5):

Exclusion criteria

1. „Have you had new serious physical illnesses since your last hospital stay?"

   ☐ unknown ☐ no ☐ yes, which one?________________________________

   In case of estimated survival <1y., then stop!

2. "Have you had any new serious mental or psychosomatic illnesses since your last hospital stay?" (Note: „serious" = e.g. acute psychosis, addictions other than tobacco)

   ☐ unknown ☐ no ☐ yes, then stop!, which one?_____________________

3. „Have you had a heart attack since your last hospital stay or did you require heart surgery?"

   ☐ unknown ☐ no ☐ yes, then stop!

4. „Are you already taking part or do you intend to take part in another treatment trial?"

   ☐ unknown ☐ no ☐ yes, which ohne?______________________________

   Then, where appropriate, stop!

Risk factors

1. „Do you smoke?"

   ☐ not specified ☐ no ☐ yes, number of cigarettes/day______

2. „How much time **per week** do you usually spend with **moderate** physical activity? (Moderate activities refer to moderate physical exertion in which you breathe a little more than normal, such as carrying light loads, cycling at normal speed or swimming at normal speed. This does not include walking at normal speed.)

   ________ minutes per week

Pat. ID:	Site Code:	Date:

3. „How much time **per week** do you usually spend on **strenuous** physical activity?" (Strenuous activities describe activities that require heavy physical exertion in which you breathe much more than normal, e.g. aerobics, running, fast cycling or fast swimming)"

   _______ minutes per week

4. Physical inactivity
   ➔ Do not ask, but calculate as follows:
   ➔ Answer Item 7 + (2x Answer Item 8)= __________ minutes

   ☐ no, if >=150 min. ☐ yes, if <150 min.

5. „Do you know your current (measurement within the last 14 days) blood pressure values?" (highest value at rest)

   ☐ no, continue with Item 7
   ☐ yes, values: _______syst.(mmHg) ________diast.(mmHg)
   → Date of measurement: _ _._ _._ _ _ _ (dd.mm.yyyy)

6. Elevated blood pressure?

   ☐ no, if BPsyst<140mmHg and BPdiast<90mmHg
   ☐ yes, if BPsyst>=140mmHg and/or BPdiast>=90mmHg
   ☐ unknown, if value is missing or too old

7. „Do you know your current (measurement within the past month) LDL cholesterol value?"

   ☐ no, continue with Item 9
   ☐ yes, value: _____(mg/dl), Date of measurement: _ _._ _._ _ _ _ (dd.mm.yyyy)

8. LDL elevated?

   ☐ no, if LDL<70mg/dl
   ☐ yes, if LDL>=70mg/dl
   ☐ unknown, if value is missing or too old

Pat. ID:	Site Code:	Date:

9. „Do you know your current (measurement within the past month) long-term blood sugar value (HbA1c)?“

☐ no, then end of interview
☐ yes, value: ________(%), Date of measurement: _ _._ _._ _ _ _ (dd.mm.yyyy)

10. HbA1c elevated?
☐ no, if HbA1c<7% or no diabetes known so far
☐ yes, if HbA1c>=7%
☐ unknown, if value is missing or too old

Notes (other, e.g. if the patient indicates that he/she is no longer interested in participating in the study)

____
____
____
____

Evaluation:

☐ No risk factor present („no“ to Items 1, 4, 6, 8 and 10)
☐ Min. 1 risk factor present („yes“ to Items 1, 4, 6, 8 or 10)
☐ Unknown (no risk factor present, but values are missing/not current or unclear)

1. BASIC DATA AND PHYSICAL EXAMINATION

1.1. Gender O male O female O diverse

1.2. Date of birth ___ . ______ mm.yyyy

1.3. Height ______ cm

1.4. Weight ______ kg

1.5. Waist circumverence ______ cm

1.6. Blood pressure (after 5 minutes of rest, 3 measurements in 2-minute intervals)

1. Value systolic ______ mmHg diastolic ______ mmHg

2. Value systolic ______ mmHg diastolic ______ mmHg

3. Value systolic ______ mmHg diastolic ______ mmHg

1.7. Heart rate (after 5 minutes of rest in sitting position) ______ bpm

Orhythmic Oarrhythmic

1.8. Menopause Oyes Ono Ounknown

1.9. Other physical findings (e.g. orthopedic/neurologic limitations)

Oyes, specify:______________________________ Ono

____

2. CARDIOVASCULAR RISK FACTORS (history and external findings)

2.1. Diabetes mellitus Oyes Ono Ounknown

2.2. Arteriel hypertension Oyes Ono Ounknown

2.3. Dyslipidemia Oyes Ono Ounknown

2.4. Smoking Oyes, no. of cigarettes/day (current) _____ Pack years ______

Ono O e-cigarettes only Ounknown

OEx-smoker, since ___ . ______ mm.yyyy

2.5. Alcoholic drinks per weeks __________
(dpw=drinks per week; 1 drink = 0.25 l beer oder 0.1 l wine oder 2 cl liquor)

Olow (< 1dpw) Omoderate (1-10 dpw) Ohigh (> 10dpw)

2.6. Familiy history of CVD **O yes** **O no** **O unknown**
(myocardial infarction or stroke in biological parents, siblings or children below 65 years of age in women and below 55 years of age in men)

If yes, who? __________ when? (Age____; Year _________)

Additional information: _______________________

3. CARDIAC DIAGNOSES (history and external findings)

3.1. Coronary heart disease O 1-vessel CHD O 2-vessel CHD O 3- vessel CHD

Last coronary angiogram, date ___ . ______ mm.jjjj

Additional information (e.g. stenoses): _______________________

CCS class O I O II O III O IV

3.2. Hx of myocardial infarction O yes O no O unknown

If yes, when (last)? _____________

3.3. Heart failure O yes O no O unknown

EF ___ % O echokardiogram O ventriculogram

NYHA class O I O II O III O IV

3.4. Atrial fibrillation / flutter O yes, current O yes, history of O no O unknown

3.5. Other cardiac diseases (cardiomyopathy, Hx of myocarditis, Hx of endocarditis, Valvular heart defects, Cardiac arrhythmias)

Please specify;____

Please specify;____

Please specify;____

4. PREVIOUS CARDIOVASCULAR INTERVENTIONS (history and external findings)

4.1. Percutaneous coronary revascularization O yes O no O unknown

If yes, when (last)? _______________

4.2. Coronary bypass surgery O yes O no O unknown

If yes, when (last)? _______________

4.3. Implanted cardiac pacemaker or defibrillator? O no O Pacemaker O Defibrillator

If yes, since when? _______________

4.3.1 If implanted defibrillator in place: DC shock delivery? O yes O no O unknown

If yes, adequate? O yes O no O unknown

If yes, when (last)? _______________

If yes, no. of shocks?_________

4.4. Other cardiovascular interventions/surgery O yes O no O unknown

If yes, specify _________________________________

If yes, when (last)? _______________

If yes, specify _________________________________

If yes, when (last)? _______________

5. COMORBIDITIES (history and external findings)

5.1. Peripheral vascular occlusive disease O yes O no O unknown

5.2. Stroke/TIA O yes O no O unknown

5.3. Chronic lung disease O yes O no O unknown

5.4. Renal insufficiency O yes O no O unknown

5.5. Current dialysis treatment O yes O no O unknown

5.6. Malignant disease <5 years ago O yes O no O unknown

If yes, specify _________________________________

5.7. Malignant disease >5 years ago Oyes Ono Ounknown

If yes, specify ___________________________________

5.8. Thyroid disease Oyes Ono Ounknown

If yes, specify ___________________________________

5.9. Inflammatory rheumatic disease Oyes Ono Ounknown

5.10. Hx. of Covid-19 infection Oyes Ono Ounknown

If yes, requiring intensive care? Oyes Ono

5.11. Other somatic comorbidities Oyes Ono Ounknown

If yes, specify ___________________________________

6. MENTAL/PSYCHOSOMATIC COMORBIDITY (history and external findings)

6.1. Depression Oyes Ono Ounknown

If yes, diagnosed by Ophysician Oself-report Ounknown

If yes, >1 episodes? Oyes Ono

If yes, when (last)? __________ (year)

6.2. Anxiety disorder Oyes Ono Ounknown

If yes, diagnosed by Ophysician Oself-report Ounknown

6.3. Somatoform disorder Oyes Ono Ounknown

If yes, diagnosed by Ophysician Oself-report Ounknown

6.4. Substance abuse / addiction Oyes Ono Ounknown

If yes, which substance(s)? ________________

If yes, diagnosed by Ophysician Oself-report Ounknown

6.5. Other diagnosen from ICD-10, chapter F Oyes Ono Ounknown

If yes, specify ___________________________________

If yes, specify ___________________________________

7. UTILIZATION OF HEALTH CARE or PSYCHOTHERAPY

7.1. Previous psychiatric treatment or psychotherapy O yes O no

If yes, when? O currently O previously

If yes, modality? O out-patient O in-patient O both

If yes, type? O psychotherapy O drug treatment O both

7.2. No. of psychotherapy sessions during last 12 months: __________ sessions

7.3. No. of psychiatric consultations during last 12 months: __________ consultations

7.4. No. of physician consultations during last 4 weeks:

O Family physician, _____ consultations

O Specialists, _____ consultations

7.5. Hospitalizations for heart disease during last 12 months:

No. __________, total no. of days _________

7.6. Cardiac rehabilitation during last 12 months:

O in-patient O out-patient O both

No. __________, total no. of days _________

7.7. Hospitalizations for mental illness during last 12 months (including in-patient psychosomatic rehabilitation):

No. __________, total no. of days _________

7.8. Hospitalizations for other reasons during last 12 months:

No. __________, total no. of days _________

7.9. Inability to work during last 12 months:

No. __________, total no. of days _________

8. DRUGS

8.1. Cardiovascular drugs (classification of current drugs)

ACE inhibitors (e.g. Ramipril) O yes O no O unknown

Angiotensin receptor blockers (e.g. Valsartan) O yes O no O unknown

Aldosterone antagonists (e.g. Spironolactone) O yes O no O unknown

Sacubitril/Valsartan (Entresto®) O yes O no O unknown

Amiodarone (e.g. Cordarex®) O yes O no O unknown

Other antiarrhythmics O yes O no O unknown

Beta blockers (e.g. Bisoprolol) O yes O no O unknown

Calcium antagonists (e.g. Amlodipine) O yes O no O unknown

Thiazides (e.g. HCT) O yes O no O unknown

Loop diuretics (e.g. Torasemide) O yes O no O unknown

Cardiac glykosides (e.g. Digoxin) O yes O no O unknown

Nitrates O yes O no O unknown

Ranolazine (e.g. Ranexa®) O yes O no O unknown

Ivabradine (e.g. Procoralan®) O yes O no O unknown

Statin (e.g. Atorvastatin) O yes O no O unknown

Other lipid lowering drugs O yes O no O unknown

8.2. Anticoagulants (classification of current drugs)

ASS O yes O no O unknown

Thienopyridines O yes O no O unknown
(e.g.. Clopidogrel, Prasugrel, Ticagrelor)

Vitamin K antagonists O yes O no O unknown
(e.g. Phenprocoumon)

Direct oral antikoagulants O yes O no O unknown
e.g. Apixaban, Edoxaban, Dabigatran)

8.3. Antidiabetics (classification of current drugs)

**Insulin**	O yes	O no	O unknown
**Oral antidiabetics** (e.g. Metformin)	O yes	O no	O unknown

8.4. Analgesics (classification of current drugs)

**NSAR** (e.g. Ibuprofen)	O yes	O no	O unknown
**Opioids** (e.g. Tramadol, Oxycodone)	O yes	O no	O unknown

8.5. Hormones (classification of current drugs)

**Thyroid medications**	O yes	O no	O unknown
**Oral contraceptives**	O yes	O no	O unknown
**Other hormones**	O yes	O no	O unknown

8.6. Psychotropics (classification of current drugs)

Antidepressants/anxiolytics

**SSRIs** (e.g. Sertraline)	O yes	O no	O unknown
**SSNRIs** (e.g. Venlafaxine)	O yes	O no	O unknown
**Tricyclics** (e.g. Amitriptyline)	O yes	O no	O unknown
**Tetracyclics** (e.g. Mirtazapine)	O yes	O no	O unknown
**MAO inhibitors** (e.g. Tranylcypromine)	O yes	O no	O unknown
**Agomelatin** (e.g. Valdoxan®)	O yes	O no	O unknown
**Others,** Specify________________	O yes	O no	O unknown

Hypnotics/Tranquilizers

**Benzodiazepines**	O ja	O no	O unknown
**Zolpidem/Zopiclon**	O ja	O no	O unknown

Others, Specify______________________

Antipsychotics/neuroleptics

Phenothiazines (e.g. Promethazine, Levomepromazine) **O**yes **O**no **O**unknown

Butyrophenones (e.g. Pipamperone, Melperone, Haloperidol) **O**yes **O**no **O**unknown

Atypical antipsychotics (e.g. Quetiapine, Risperidone) **O**yes **O**no **O**unknown

Others, Specify______________________________

8.7. Other drugs (not to be classified above)

Please specify ______________________________________

Please specify ______________________________________

Please specify ______________________________________

Please specify ______________________________________

Please specify ______________________________________

Please specify ______________________________________

Please specify ______________________________________

(Dietary habits will be assessed by the German version of the Mediterranean Diet Adherence Screener [MEDAS]: *Hebestreit K, Yahiaoui-Doktor M, Engel C, Vetter W, Siniatchkin M, Erickson N, Halle M, Kiechle M, Bischoff SC. Validation of the German version of the Mediterranean Diet Adherence Screener (MEDAS) questionnaire. BMC Cancer. 2017;17:341.****)***

(Psychometric scales will include the HeartQoL, HADS, and PSS-4 as well as additional instruments listed in Table 4 of the main manuscript)

	TEACH – Team-based treatment of coronary heart disease<br><br>BASELINE-ASSESSMENT	Patient-ID:<br><br>Date:
TEACH	**SOCIO-DEMOGRAPHIC INFORMATION**<br>**Self-evaluation questionnaire**	

1. What is your marital status? Please check one that applies to you.

- O Married/registered civil partnership
  - O and living with my partner in one household
  - O and not living with my partner in one household
  - O and being separated without current partnership

- O Not-married, no registered civil partnership
- O Divorced
- O Widowed
  - O and living with my partner in one household
  - O and having a partnership, but not a common household
  - O and without current partnership

2. What is your highest graduation certificate?

(Please check only the highest degree)

- o No graduation
- o Secondary school qualification or equivalent qualification
- o Secondary modern school qualification (medium maturity) or equivalent qualification
- o Advanced college certificate
- o High school graduation
- o Other kind of graduation: ______________________________

3. What professional qualifications do you have?
(Please check only the highest degree)

- o No professional qualification
- o Apprenticeship / vocational school / technical school completed
- o Master's school, technical school, administrative and business academy or university of applied sciences completed
- o Bachelor
- o University diploma, master, magister, state examination, doctorate
- o Other kind of qualification: ______________________________

4. What is your current employment?

(Please note that employment means any paid or income-related activity)

o Full-time employment

o Part-time employment

o Partial retirement

o Mini job (up to 450 Euro/months)

o "One euro job" (and receiving unemployment benefit)

o Irregularly employed

o On educational leave (and receiving salary)

o Maternity/parental leave or other leave or absence

o Not employed (unemployed, early retirees, retirees without additional salary)

5. What professional position do you have or did you have in your main job?

- Self-employed business owner (farmer, in trade, the hospitality industry, handicraft, industry, service)
- Self-employed academic (physician, lawyer, tax consultant, etc.)
- Civil servant, judge or professional soldier
- White collar employee
- Blue collar worker
- Helping family members

6. If you are not working full-time or part-time: Please tell us which group you belong to on this list.

- Pensioners, retirees, in early retirement
- Unemployed
- Permanently disabled
- Housewives/Housemen
- Other:______________________________

7. What is the total average monthly <u>net income</u> of your household?
The average monthly net income of your household is comprised as follows: Combine all monthly wages, salaries, income from self-employment or pensions from your household (this may also include your spouse). Please also add the income from public subsidies, income from renting and leasing, assets, housing benefit, child benefit and other income on a monthly basis. Then deduct taxes and social security contributions. The result is your household net income.
.

- ○ Less than 850 Euros
- ○ 851 to less than 1 500 Euros
- ○ 1 500 to less than 2 500 Euros
- ○ 2 500 to less than 3 500 Euros
- ○ 3 500 to less than 4 500 Euros
- ○ 4500 Euros or more
- ○ Not specified

8. Did you apply for a pension or social security? O no O yes

If yes, has it already been approved? O no O yes, since? ___ . ____ mm.yyyy

9. What health insurance do you belong to? O statutory O private O social welfare office

TEACH Team-basierte Behandlung der Coronaren Herzkrankheit

T6 – Telephone assesment 6 months after V0

Pat. ID:	Site Code:	Date:

Part A. SOCIO-DEMOGRAPHIC INFORMATION

1. „Has anything changed in your marital status in the **past 6 months**?"
   - O no
   - O yes → „What has changed?"
     - ☐ Partner died
     - ☐ Seperated or divorced from partner
     - ☐ New registered civil partnership or newly married →
       - ☐ and live with my partner in one household
       - ☐ but not a common household

Part B. CARDIOVASCULAR RISK FACTORS

2. Only if „no/unknown" was answered in V0, Item 2.1: "Have you been diagnosed with diabetes mellitus in the meantime?"
   O yes O no O unknown

3. Only if „no/unknown" was answered in V0, Item 2.2: " Have you been diagnosed with arterial hypertension (high blood pressure) in the meantime?"
   O yes O no O unknown

4. "Have you consumed nicotine in the past 6 months?"
   O no O unknown O Ex-smoker, since ___ . ______ mm.yyyy

   O yes → O only E-cigarettes → "How many cigarettes do you smoke currently per day (incl. E-cigarettes)?"______

5. "How many glasses of alcohol do you drink per week?"

   (1 glass= 0,25l beer or 0,1l wine or 2cl liquor) _________(number)

   O low (<1 glass/week)

   O moderate (approx. 1-10 glasses/week)

   O high (>10 glasses/week)

Pat. ID:	Site Code:	Date:

Part C. DIAGNOSES

6. "Have you had any new heart disease (myocardial infarction, heart failure, cardiac arrhythmias, fainting spells, other heart disease) in the past 6 months?"
   O yes → O no
   - ☐ Myocardial infarction
   - ☐ Heart failure
   - ☐ Cardiac arrhythmias
   - ☐ Fainting spells
   - ☐ Other heart disease, which one?______________________________

7. "Have you had any new mental/psychosomatic illnesses in the past 6 months?"
   O yes, which one?_____________________________ O no

8. "Have you had any new physical illnesses in the past 6 months?"
   O yes, which one?_____________________________ O no

Part D. CARDIOVASCULAR INTERVENTIONS

9. "Have you had any heart surgery/interventions in the past 6 months?"
   O yes → O no
   - ☐ Percutaneous coronary revascularization
   - ☐ Coronary bypass surgery
   - ☐ Implanted cardiac pacemaker
   - ☐ Other cardiovascular interventions/surgery, which one?____
   - ☐ Implanted defibrillator
     → „DC shock delivery?"
     O yes O no O unknown
     → If yes, adequate? O yes O no O unknown
     Number of shocks?_____________

Pat. ID:	Site Code:	Date:

Part E. UTILIZATION OF HEALTH CARE or PSYCHOTHERAPY

10. "How many physician consultations have you had in the past 6 months?"
   O Family physician,____consultations O Specialist,_____consultations

11. "Have you been hospitalized in the past 6 months?"
   O yes O no
   → "Due to which disease, how often and for how long?"
   - ☐ Heart disease, Number______, total number of days______
   - ☐ Mental illness (including in-patient psychosomatic rehabilitation), Number______, total number of days______
   - ☐ Other reasons, Number______, total number of days______

Part F. SAE

12. „Have you attempted to commit suicide in the past 6 months?"
   O yes O no

Part G. DRUGS

13. "Has anything changed in your medication in the past 6 months?"
   O yes O nein
   → "What medication are you currently taking?"
   (List of current medications same as baseline)

Part H. PSYCHOMETRIC ASSESSMENT

(Psychometric scales include HeartQoL, HADS, and PSS-4)

TEACH
Team-basierte Behandlung der Coronaren Herzkrankheit

V12 – In-person visit 12 months after V0

Pat. ID:	Site Code:	Date:

Part A. SOCIO-DEMOGRAPHIC INFORMATION

1. „Has anything changed in your marital status in the **past 6 months**?"
   ○ no ○ yes → „What has changed?"
   - ☐ Partner died
   - ☐ Separated or divorced from partner
   - ☐ New registered civil partnership or newly married
     - ☐ and live with my partner in one household
     - ☐ but not a common household

2. "Has anything changed in you employment situation in the **past year**?"
   ☐ no ☐ yes → ***(employment status same items as baseline)***

3. Only if „no/unknown" was answered in V0, Item 8: "Did you apply for a pension or social security during the last year?"
   ☐ no ☐ yes → "Has it already been approved and since when?"
   ○ no ○ yes, since ___ . _____ mm.yyyy

Part B. PHYSICAL EXAMINATION

4. **Weight** _______ kg
5. **Waist circumference** _______ cm
6. **Blood pressure (*same as baseline*)**
7. **Heart rate (*same as baseline*)**
8. Only for women if „no/unknown" was answered in V0 Item1.9: **Menopause**
   ☐ yes ☐ no ☐ unknown
9. **Other physical findings (*same as baseline*)**

V12 – In-person visit 12 months after V0

Pat. ID:	Site Code:	Date:

Part C. CARDIOVASCULAR RISK FACTORS

10. Only if „no/unknown“ was answered in T6, Item 2. "Have you been diagnosed with diabetes mellitus in the meantime?"
O yes   O no   O unknown

11. Only if „no/unknown“ was answered in T6, Item 3: " Have you been diagnosed with arterial hypertension (high blood pressure) in the meantime?”
O yes   O no   O unknown

12. Only if „no/unknown“ was answered in T6, Item 4: " Have you been diagnosed with dyslipidemia in the meantime?"
O yes   O no   O unknown

13. "Have you consumed nicotine in the past 6 months?" (***same as T6***)

14. **Alcoholic drinks per weeks** (***same as baseline***)

Part D. DIAGNOSES (***same as T6***)

Part E. CARDIOVASCULAR INTERVENTIONS (***same as T6***)

Part F. UTILIZATION OF HEALTH CARE or PSYCHOTHERAPY

19. "Have you received psychiatric or psychotherapeutic treatment in the **past year**?"
O yes   O no
→ ***(same options as baseline)***

20. "How many physician consultations have you had in the **past 6 months**?"
O Family physician,_____consultations   O Specialist,______consultations

21. "Have you been hospitalized in the past 6 months?"
O yes   O no
→ ***(same options as T6)***

22. "Have you participated in cardiac rehabilitation during the **past year**?"
O yes   O no
→ „What kind of modality, how often and for how long?"

O out-patient   O in-patient   O both

Number______, total number of days_____

23. "Have you been unable to work during the past year?“

O yes   O no

→ „How often and for how long?"

Number______, total number of days_____

Part G. SAE

24. “Have how attempted suicide during the **past 6 months**?”

O yes   O no

Part H. DRUGS

25. "Has anything changed in your medication in the past 6 months?"

O yes   O nein

→ "What medication are you currently taking?"

(List of current medications same as baseline)

Part I. DIET ***(same as baseline)***

Part J. PSYCHOMETRIC ASSESSMENT ***(same as baseline)***

TEACH
Team-basierte Behandlung der Coronaren Herzkrankheit

V18– In-person visit 18 months after V0

Pat. ID:	Site Code:	Date:

Part A. SOCIO-DEMOGRAPHIC INFORMATION

1. „Has anything changed in your marital status in the **past 6 months**?“ ***(same as V12)***

2. "Has anything changed in you employment situation in the **past 6 months**?"
   ☐ no ☐ yes
   → ***(employment status same items as baseline)***

3. Only if „no/unknown“ was answered in V12, Item 3: "Did you apply for a pension or social security during the last six months?" ***(same options as V12)***

Part B. PHYSICAL EXAMINATION ***(same as V12)***

Part C. CARDIOVASCULAR RISK FACTORS

10. Only if „no/unknown“ was answered in V12, Item 10: "Have you been diagnosed with diabetes mellitus in the meantime?"
    O yes O no O unknown

11. Only if „no/unknown“ was answered in V12, Item 11: " Have you been diagnosed with arterial hypertension (high blood pressure) in the meantime?”
    O yes O no O unknown

12. Only if „no/unknown“ was answered in V12, Item 12: "Have you been diagnosed with dyslipidemia in the meantime?"
    O yes O no O unknown

13. "Have you consumed nicotine in the past 6 months***?" (same as V12)***

14. **Alcoholic drinks per weeks *(same as V12)***

Part D. DIAGNOSES ***(same as T6)***

Part E. CARDIOVASCULAR INTERVENTIONS (***same as T6)***

Part F. UTILIZATION OF HEALTH CARE or PSYCHOTHERAPY

19. "Have you received psychiatric or psychotherapeutic treatment in the **past 6 months**?"
    O yes O no
    → ***(same options as baseline)***

20. "How many physician consultations have you had in the **past 6 months**?"
   O Family physician,____consultations   O Specialist,_____consultations

21. "Have you been hospitalized in the past 6 months?"
   O yes   O no
   → ***(same options as V12)***

22. "Have you participated in cardiac rehabilitation during the **past 6 months**?"
   O yes   O no
   → ***(same options as T6)***

23. "Have you been unable to work during the past 6 months?"
   O yes   O no
   → ***(same options as T6)***

Part G. SAE

24. "Have you attempted suicide during the **past 6 months**?"
   O yes   O no

Part H. DRUGS

25. "Has anything changed in your medication in the past 6 months?"
   O yes   O no
   → "What medication are you currently taking?"
   (List of current medications same as baseline)

Part I. DIET ***(same as baseline)***

Part J. PSYCHOMETRIC ASSESSMENT ***(same as baseline)***

T24/T30 – Telephone assessment 24/30 months after V0

Pat. ID:	Site Code:	Date:

Part A. DIAGNOSES (same as T6)

Part B. CARDIOVASCULAR INTERVENTIONS *(same as T6)*

Part C. SAE *(same as T6)*

Part D. DRUGS *(same as T6)*

Part E. PSYCHOMETRIC ASSESSMENT *(same as T6)*

(If this is the last assessment, additional information will be obtained according to V12 on

- **SOCIODEMOGRAPHICS**
- **CARDIOVASCULAR RISK FACTORS**
- **UTILIZATION)**
